# Elevated urinary angiotensinogen excretion links central and renal hemodynamic alterations

**DOI:** 10.1038/s41598-023-38507-w

**Published:** 2023-07-17

**Authors:** Keisei Kosaki, Jiyeon Park, Masahiro Matsui, Takeshi Sugaya, Makoto Kuro-o, Chie Saito, Kunihiro Yamagata, Seiji Maeda

**Affiliations:** 1 Institute of Health and Sport Sciences, University of Tsukuba, 1-1-1 Tennodai, Tsukuba, Ibaraki 305-8574 Japan; 2 Advanced Research Initiative for Human High Performance (ARIHHP), University of Tsukuba, Ibaraki, Japan; 3 Division of Nephrology and Hypertension, Department of Internal Medicine, St. Marianna University School of Medicine, Kanagawa, Japan; 4 Division of Anti-aging Medicine, Center for Molecular Medicine, Jichi Medical University, Tochigi, Japan; 5 Department of Nephrology, Institute of Medicine, University of Tsukuba, Ibaraki, Japan; 6 R&D Center for Smart Wellness City Policies, University of Tsukuba, Tokyo, Japan; 7 Faculty of Sport Sciences, Waseda University, Saitama, Japan

**Keywords:** Predictive markers, Nephrons

## Abstract

Inappropriate activation of intrarenal renin–angiotensin system (RAS) may contribute to the pathogenesis of cardio-renal syndrome (CRS). We aimed to examine the cross-sectional associations of urinary angiotensinogen (AGT) excretion, a biomarker of intrarenal RAS activity, with central (aortic) and renal hemodynamic parameters in middle-aged and older adults, including patients with chronic kidney disease. Aortic and renal hemodynamic parameters were measured using applanation tonometry and duplex ultrasonography in 282 participants. Urinary AGT, liver-type fatty acid-binding protein (L-FABP), and plasma N-terminal pro-B-type natriuretic peptide (NT-proBNP) levels were measured for each participant. Multiple linear regression analyses demonstrated that urinary AGT levels were associated with aortic blood pressures, pulsatile measures of renal blood flow, plasma NT-proBNP and urinary L-FABP levels after adjusting for potential covariates, including age, sex, body mass index, estimated glomerular filtration rate (GFR), and medication use. Additionally, when classified based on GFR stages and urinary AGT levels, plasma NT-proBNP and urinary L-FABP levels increased in participants with lower GFR and higher AGT groups. Our findings suggest that urinary AGT excretion is a shared determinant of central (aortic) and renal hemodynamics in middle-aged and older adults, providing clinical evidence for the potential role of intrarenal RAS activity in the development of CRS.

## Introduction

Accumulating clinical evidence indicates that patients with chronic kidney disease (CKD) are at a greater risk of cardiovascular complications^1^. Conversely, cardiovascular dysfunction (e.g., heart failure) is a well-known risk factor for developing renal dysfunction^2^. These spectrum disorders involving the heart and kidneys have been widely recognized as a cardio-renal syndrome (CRS), and the complex nature of their treatment and management pose major global public health challenges. With the growing epidemic of CRS, there is an urgent need for further research to explore common predictors in these organs.

The heart and kidneys are affected by humoral, neural, and hemodynamic factors, and abnormal alterations in these factors eventually lead to the development of CRS^3,4^. Previous studies have shown that elevated renal flow pulsatility—assessed using renal duplex ultrasonography or phase-contrast magnetic resonance imaging—strongly correlates with increased aortic pulse pressure and stiffness in various populations^5–7^. Moreover, increased retrograde flow from the descending thoracic aorta toward the aortic arch has been independently associated with reduced antegrade flow into the kidneys in patients with hypertension^8^. It has been demonstrated that these hemodynamic alternations, simultaneously observed in the aorta and kidney, could contribute to the pathogenesis of concomitant plasma B-type natriuretic peptide elevation with (micro)albuminuria^9^. Collectively, these close associations between central (aortic) and renal hemodynamics have been postulated as potential mechanisms that account for the bidirectional nature of CRS.

The renin–angiotensin system (RAS) is an essential hormonal system that regulates blood pressure and fluid/electrolyte homeostasis. In addition to the well-known systemic RAS, growing evidence has demonstrated that a completely functional RAS is present along the nephron, and inappropriate activation of the intrarenal RAS is recognized as the principal pathogenesis of hypertension and CKD^10–13^. Basic evidence also indicates that intrarenal RAS activity is a crucial mediator of CRS^14^. In this context, recent studies have reported that measuring the urinary levels of angiotensinogen (AGT), a substrate for renin, allows for the estimation of intrarenal RAS activity in patients with hypertension^15,16^ and CKD^17–19^. Moreover, a recent scientific statement from the American Heart Association (AHA) has proposed urinary AGT excretion as a diagnostic and prognostic biomarker of CRS^2^. However, to date, clinical evidence supporting the use of urinary AGT excretion as a biomarker for CRS has not been fully elucidated.

Based on this background, we hypothesized that augmented intrarenal RAS activity links to both central (aortic) and renal hemodynamic alterations. To test this hypothesis, we investigated the cross-sectional associations of urinary AGT levels with central (aortic) and renal hemodynamic parameters in middle-aged and older adults, including patients with CKD. Clarifying whether urinary AGT excretion is a shared determinant of central (aortic) and renal hemodynamics may lead to a better understanding of the potential role of intrarenal RAS activity in the development of CRS.

## Results

Table 1 summarizes the clinical characteristics classified according to the estimated glomerular filtration rate (GFR). Based on the group comparison of GFR stages, participants in the lower GFR groups (60–89 or < 59 mL/min/1.73 m^2^) were older; had a lower proportion of females; had a higher percentage of antihypertensive (i.e., renin–angiotensin system inhibitor, calcium channel blocker, diuretic, β-blocker), lipid-lowering, and glucose-lowering medication users; had lower high-density lipoprotein (HDL) cholesterol and low-density lipoprotein (LDL) cholesterol levels; and had higher height, weight, body mass index (BMI), triglyceride, and hemoglobin A1c levels, mean arterial pressure, and brachial systolic blood pressure (SBP) and diastolic blood pressure (DBP).

**Table 1:** Clinical characteristics according to estimated GFR levels.

Variables	GFR stages (mL/min/1.73 m^2^)						*P*-value
	≥ 90		60–89		< 60		
n	101		121		60		**–**
Age (years)	58 [54–65]		68 [62–73]		67 [58–73]		< 0.001
Women, n (%)	90 (89)		71 (59)		25 (42)		< 0.001
Height (cm)	156 [152–162]		159 [153–167]		161 [154–167]		0.017
Weight (kg)	52 [45–61]		58 [50–65]		61 [54–68]		< 0.001
Body mass index (kg/m^2^)	21.1 [19.3–23.8]		22.5 [20.6–24.4]		23.5 [20.7–25.8]		0.002
High-density lipoprotein cholesterol (mg/dL)	75 [63–88]		65 [53–83]		65 [50–73]		< 0.001
Low-density lipoprotein cholesterol (mg/dL)	125 [111–144]		129 [109–153]		110 [95–125]		< 0.001
Triglyceride (mg/dL)	74 [54–92]		91 [70–122]		102 [72–137]		< 0.001
Fasting plasma glucose (mg/dL)	99 [93–106]		100 [95–109]		102 [95–112]		0.091
Hemoglobin A1c (mg/dL)	5.7 [5.5–5.9]		5.7 [5.5–6.0]		5.9 [5.6–6.2]		0.009
Heart rate (bpm)	60 [53–64]		59 [55–64]		62 [55–68]		0.258
Mean arterial pressure (mmHg)	85 [78–91]		91 [84–99]		94 [88–101]		< 0.001
Brachial systolic blood pressure (mmHg)	116 [108–126]		124 [114–134]		125 [117–137]		< 0.001
Brachial diastolic blood pressure (mmHg)	69 [64–75]		76 [68–82]		78 [73–83]		< 0.001
Brachial pulse pressure (mmHg)	47 [41–53]		49 [43–56]		47 [43–57]		0.333
Renin–angiotensin system inhibitor use, n (%)	3 (3)		21 (17)		41 (68)		< 0.001
Calcium channel blocker use, n (%)	13 (13)		15 (12)		25 (42)		< 0.001
Diuretic use, n (%)	1 (1)		3 (2)		6 (10)		0.008
β-blocker use, n (%)	1 (1)		4 (3)		7 (12)		0.004
Lipid-lowering use, n (%)	11 (11)		26 (21)		35 (58)		< 0.001
Glucose-lowering use, n (%)	3 (3)		3 (2)		13 (22)		< 0.001
Anti-hypertensive use, n (%)	15 (15)		28 (23)		48 (80)		< 0.001

Values are presented as the median [interquartile range] or frequency counts (%). *GFR* glomerular filtration rate.

The central (aortic) and renal characteristics according to GFR stages are shown in Table 2. Compared with the normal GFR group (≥ 90 mL/min/1.73 m^2^), the lower GFR groups (60 to 89 or < 59 mL/min/1.73 m^2^) exhibited higher levels of aortic SBP and DBP, carotid-femoral pulse wave velocity (PWV), plasma N-terminal pro-B-type natriuretic peptide (NT-proBNP), renal resistive index (RI) and pulsatility index (PI), serum fibroblast growth factor 23 (FGF23), urinary albumin creatinine ratio (ACR), β2-microglobulin, urinary liver-type fatty acid-binding protein (L-FABP), and urinary AGT excretion.

**Table 2:** Central (aortic) and renal characteristics according to estimated GFR levels.

Variable	GFR stages (mL/min/1.73m^2^)						*P* value
	≥ 90		60–89		< 60		
Central (aortic) measures							
Aortic systolic blood pressure (mmHg)	106 [98–113]		113 [103–122]		115 [107–126]		< 0.001
Aortic diastolic blood pressure (mmHg)	69 [64–75]		76 [68–82]		78 [73–83]		< 0.001
Aortic pulse pressure (mmHg)	36 [31–42]		38 [33–43]		37 [33–46]		0.175
Aortic incident wave height (mmHg)	21 [18–25]		22 [19–27]		24 [19–27]		0.163
Aortic augmented pressure (mmHg)	11 [8–14]		11 [8–14]		12 [9–14]		0.451
Carotid-femoral pulse wave velocity (cm/s)	715 [641–809]		825 [740–951]		871 [807–1069]		< 0.001
Plasma NT-proBNP (pg/mL)	51 [34–66]		53 [33–98]		71 [36–137]		0.024
Renal measures							
Renal resistive index	0.60 [0.57–0.63]		0.62 [0.59–0.67]		0.66 [0.62–0.70]		< 0.001
Renal pulsatility index	1.00 [0.92–1.08]		1.07 [0.98–1.21]		1.18 [1.05–1.31]		< 0.001
Serum FGF23 (pg/dL)	39 [31–48]		45 [35–59]		72 [54–102]		< 0.001
Estimated GFR (mL/min/1.73m^2^)	97 [94–105]		79 [72–83]		46 [38–56]		< 0.001
Urinary albumin creatinine ratio (mg/g)	10 [7–19]		12 [7–31]		115 [24–983]		< 0.001
Urinary β2-microglobulin (μg/g Cr)	173 [114–289]		172 [114–278]		304 [99–2198]		0.006
Urinary L-FABP (μg/g Cr)	1.2 [0.5–2.2]		1.1 [0.4–2.1]		4.6 [1.4–13.9]		< 0.001
Urinary angiotensinogen (μg/g Cr)	9.0 [4.4–17.7]		12.3 [6.5–25.8]		42.9 [9.9–212.3]		< 0.001

Values are presented as the median [interquartile range] *NT-proBNP* N-terminal pro-B-type natriuretic peptide, *FGF23* fibroblast growth factor 23, *GFR* glomerular filtration rate, *L-FABP* liver-type fatty acid-binding protein.

**Figure 1:**
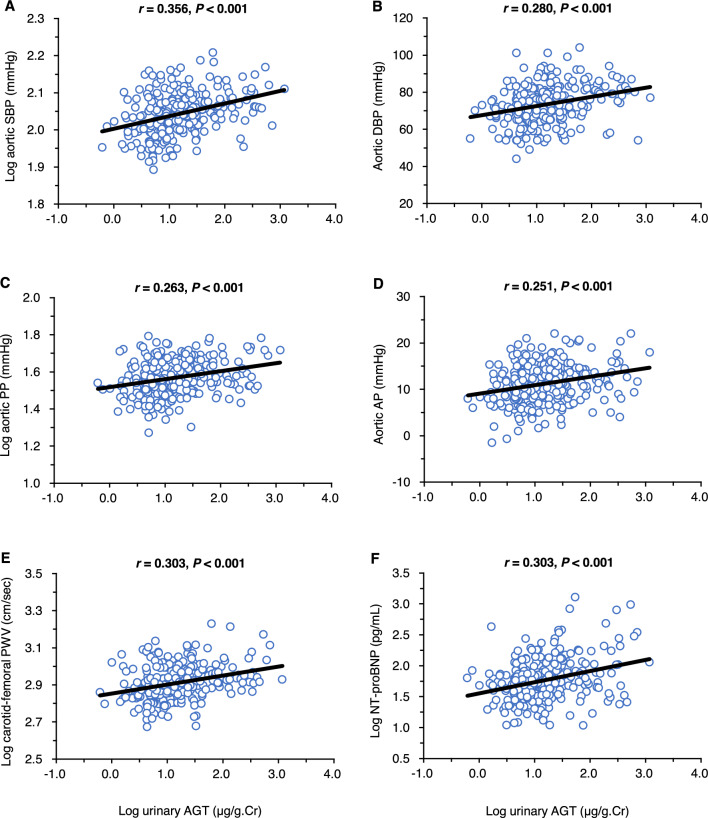
Simple correlations between urinary angiotensinogen levels and central (aortic) hemodynamic parameters.

**Figure 2:**
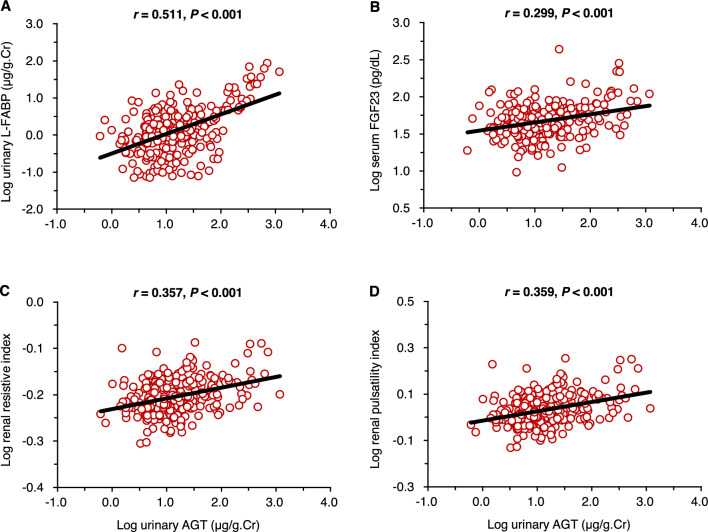
Simple correlations between urinary angiotensinogen levels and renal hemodynamic parameters.

Figure 1 shows the simple correlations of urinary AGT levels with central (aortic) hemodynamic parameters. Positive correlations were observed between urinary AGT levels and aortic SBP (panel A), DBP (panel B), pulse pressure (panel C), augmented pressure (panel D), carotid-femoral PWV (panel E), and plasma NT-proBNP levels (panel F). Figure 2 shows the simple correlations of urinary AGT levels with renal hemodynamic parameters. The results revealed that urinary AGT levels were closely and positively correlated with urinary L-FABP levels (panel A), serum FGF23 levels (panel B), and renal RI (panel C) and PI (panel D) values.

Table S1 presents the simple correlation matrix for urinary AGT and β2-microglobulin levels in individuals with and without albuminuria. Regardless of the presence or absence of albuminuria, we found a positive correlation of urinary AGT and β2-microglobulin levels with plasma NT-proBNP and urinary L-FABP levels, and urinary ACR. Furthermore, in individuals with normoalbuminuria, we observed a positive correlation between urinary AGT levels and aortic SBP and DBP, carotid-femoral PWV, and renal RI and PI. However, we did not find a significant correlation between urinary β2-microglobulin levels and these parameters.

Table S2 presents the simple correlation matrix for urinary AGT and β2-microglobulin levels in individuals with higher and lower plasma NT-proBNP levels. Regardless of the plasma NT-proBNP levels, urinary AGT levels were positively correlated with aortic SBP and DBP, carotid-femoral PWV, urinary L-FABP levels, urinary ACR, renal RI and PI, and serum FGF23 levels.

The multiple adjusted associations of urinary AGT levels with central (aortic) and renal hemodynamic parameters are presented in Tables 3 and 4. Excluding carotid-femoral PWV, the associations between urinary AGT levels and central (aortic) hemodynamic parameters remained significant after adjusting for potential covariates, including age, sex, BMI, estimated GFR, and medication use (Table 3). Similarly, excluding serum FGF23 levels, urinary AGT levels were found to be independent determinants of renal hemodynamic parameters after considering various potentially relevant factors (Table 4).

**Table 3:** Multivariate-adjusted associations between urinary angiotensinogen with central (aortic) hemodynamic parameters.

Variable	B ± SE	*β*	*P*-value
Dependent variable: aortic systolic blood pressure* (overall model R^2^ = 0.265, *P* < 0.001)			
Urinary angiotensinogen levels, μg/g*	0.021 ± 0.005	0.219	< 0.001
Body mass index, kg/m^2^	0.004 ± 0.001	0.236	< 0.001
Age, years	0.001 ± 0.0003	0.210	< 0.001
Calcium channel blocker use (yes)	0.023 ± 0.008	0.163	0.004
Dependent variable: aortic diastolic blood pressure (overall model R^2^ = 0.271, *P* < 0.001)			
Sex (woman)	− 5.796 ± 1.179	− 0.265	< 0.001
Body mass index, kg/m^2^	0.746 ± 0.171	0.238	< 0.001
Urinary angiotensinogen levels, μg/g*	3.515 ± 0.969	0.198	< 0.001
Glucose-lowering use (yes)	− 5.751 ± 2.169	− 0.139	0.008
Calcium channel blocker use (yes)	3.194 ± 1.483	0.121	0.032
Dependent variable: aortic pulse pressure* (overall model R^2^ = 0.193, *P* < 0.001)			
Age, years	0.004 ± 0.001	0.320	< 0.001
Urinary angiotensinogen levels, μg/g*	0.028 ± 0.009	0.171	0.003
Sex (woman)	0.037 ± 0.011	0.183	0.001
Calcium channel blocker use (yes)	0.033 ± 0.014	0.136	0.020
Dependent variable: aortic augmented pressure (overall model R^2^ = 0.304, *P* < 0.001)			
Sex (woman)	3.725 ± 0.458	0.415	< 0.001
Age, years	0.177 ± 0.026	0.360	< 0.001
Urinary angiotensinogen levels, μg/g*	1.524 ± 0.372	0.209	< 0.001
Dependent variable: carotid-femoral pulse wave velocity* (overall model R^2^ = 0.344, *P* < 0.001)			
Age, years	0.004 ± 0.001	0.401	< 0.001
Estimated glomerular filtration rate, mL/min/1.73 m^2^*	–0.140 ± 0.032	− 0.227	< 0.001
Glucose-lowering use (yes)	0.061 ± 0.019	0.166	0.001
Body mass index, kg/m^2^	0.004 ± 0.001	0.133	0.009
Dependent variable: plasma N-terminal pro-B-type natriuretic peptide* (overall model R^2^ = 0.267, *P* < 0.001)			
Urinary angiotensinogen levels, μg/g*	0.103 ± 0.038	0.174	0.006
β-blocker use (yes)	0.414 ± 0.092	0.241	< 0.001
Age, years	0.010 ± 0.002	0.237	< 0.001
Sex (woman)	0.148 ± 0.042	0.203	< 0.001
Estimated glomerular filtration rate, mL/min/1.73 m^2^*	–0.594 ± 0.159	− 0.257	< 0.001
Body mass index, kg/m^2^	–0.013 ± 0.006	− 0.125	0.024
Lipid-lowering use (yes)	–0.094 ± 0.047	− 0.119	0.044

*Log-transformed. B and *β* indicate unstandardized and standardized regression coefficients, respectively. Covariates were entered by stepwise procedure from age, sex, body mass index, estimated glomerular filtration rate, renin–angiotensin system inhibitor, calcium channel blocker, diuretics, β-blocker use, lipid-lowering and glucose lowering use.

**Table 4:** Multivariate-adjusted associations between urinary angiotensinogen with renal hemodynamic parameters.

Variable	B ± SE	*Β*	*P* value
Dependent variable: urinary liver-type fatty acid-binding protein* (overall model R^2^ = 0.335, *P* < 0.001)			
Urinary angiotensinogen levels, μg/g*	0.375 ± 0.061	0.363	< 0.001
Estimated glomerular filtration rate, mL/min/1.73 m^2^*	− 1.253 ± 0.235	− 0.312	< 0.001
Age, years	− 0.007 ± 0.004	− 0.103	0.041
Dependent variable: serum fibroblast growth factor 23* (overall model R^2^ = 0.422, *P* < 0.001)			
Estimated glomerular filtration rate, mL/min/1.73 m^2^*	− 0.829 ± 0.069	− 0.575	< 0.001
Body mass index, kg/m^2^	0.009 ± 0.003	0.140	0.003
β-Blocker use (yes)	0.106 ± 0.050	0.099	0.034
Dependent variable: renal resistive index* (overall model R^2^ = 0.429, *P* < 0.001)			
Age, years	0.002 ± 0.0002	0.410	< 0.001
Estimated glomerular filtration rate, mL/min/1.73 m^2^*	− 0.055 ± 0.014	− 0.219	< 0.001
Glucose-lowering use (yes)	0.039 ± 0.007	0.258	< 0.001
Urinary angiotensinogen levels, μg/g*	0.007 ± 0.004	0.115	0.036
Dependent variable: renal pulsatility index* (overall model R^2^ = 0.436, *P* < 0.001)			
Age, years	0.003 ± 0.000	0.406	< 0.001
Estimated glomerular filtration rate, mL/min/1.73 m^2^*	− 0.100 ± 0.024	− 0.228	< 0.001
Glucose-lowering use (yes)	0.070 ± 0.012	0.266	< 0.001
Urinary angiotensinogen levels, μg/g*	0.013 ± 0.006	0.111	0.040

*Log-transformed. B and *β* indicate unstandardized and standardized regression coefficients, respectively. Covariates were entered by stepwise procedure from age, sex, body mass index, estimated glomerular filtration rate, renin–angiotensin system inhibitor, calcium channel blocker, diuretics, β-blocker use, lipid-lowering and glucose lowering use.

**Figure 3:**
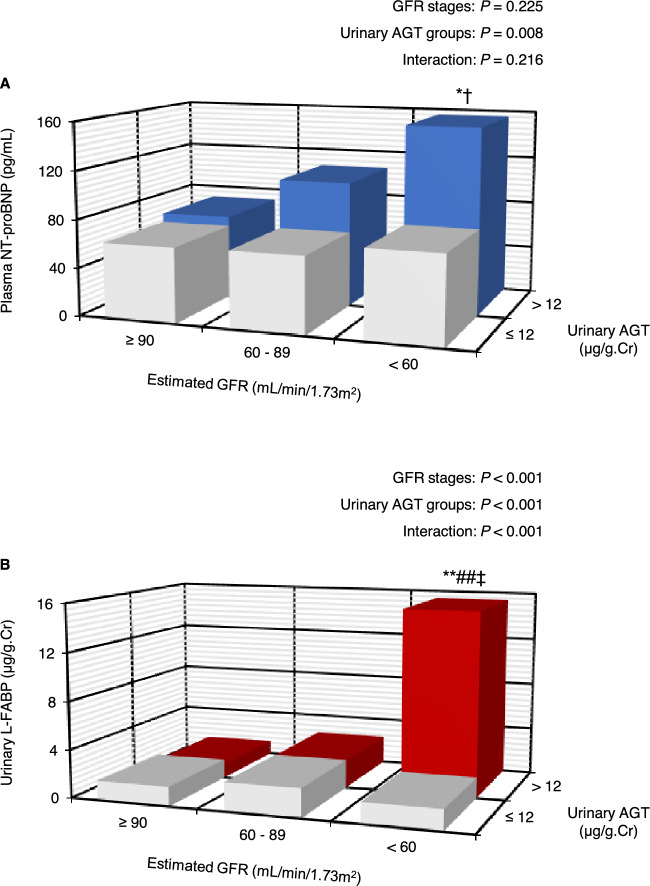
Plasma N-terminal pro-B-type natriuretic peptide (top) and urinary liver-type fatty acid-binding protein (bottom) levels in subgroups classified according to urinary angiotensinogen and estimated glomerular filtration rate levels. *P* values were calculated by analysis of covariance after adjusting for age, sex, body mass index, renin–angiotensin system inhibitor, calcium channel blocker, diuretics, β-blocker use, lipid-lowering and glucose-lowering use. **P < 0.001, *P < 0.05 vs. estimated GFR ≥ 90 mL/min/1.73 m^2^ group; ^##^*P* < 0.001 vs. estimated GFR 60–80 mL/min/1.73 m^2^ group; ^‡^*P* < 0.001, ^†^*P* < 0.001 vs. urinary AGT ≤ 12 μg/g Cr group. Multiple pairwise comparisons were corrected by the Bonferroni method.

Figure 3 shows the combined effects of urinary AGT levels and GFR stages on plasma NT-proBNP (panel A) and urinary L-FABP (panel B) levels. In the two-way analysis of covariance, significant main effects of urinary AGT groups were confirmed when participants were classified into six groups according to the three GFR stages and two urinary AGT levels. Moreover, there was a significant interaction effect of GFR stages and urinary AGT groups on urinary L-FABP levels. Increased plasma NT-proBNP and urinary L-FABP levels according to reduced renal function were only observed in the groups with higher urinary AGT levels. The mean plasma NT-proBNP (155 pg/mL) and urinary L-FABP (15.4 μg/g Cr) values were highest in the lower GFR and higher AGT groups.

## Discussion

In this study, we examined the cross-sectional associations between urinary AGT levels and several hemodynamic parameters in middle-aged and older adults, including patients with CKD. The main findings of this study were as follows: first, urinary AGT levels positively correlated with aortic blood pressures, aortic PWV, and plasma NT-proBNP levels. Moreover, urinary AGT levels positively correlated with urinary L-FABP and serum FGF23 levels, and pulsatile measures of renal blood flow, as assessed by PI and RI. Second, excluding aortic PWV and serum FGF23 levels, these associations remained significant after adjusting for potential covariates, including age, sex, BMI, estimated GFR, and medication use. Third, when participants were classified based on estimated GFR and urinary AGT levels, plasma NT-proBNP and urinary L-FABP levels increased in the group with a lower GFR and higher urinary AGT levels. Collectively, these findings significantly extend our prior knowledge of intrarenal RAS activity in humans, suggesting that urinary AGT excretion is a shared determinant of central (aortic) and renal hemodynamics.

Evidence that urinary AGT excretion can provide specific information regarding intrarenal RAS activity^20^, as well as the development of a sensitive and specific quantification system for human AGT using sandwich ELISA^21^, have contributed to the elucidation of the pathophysiological role of intrarenal RAS activity in humans^13^. Since then, growing clinical evidence has indicated that urinary AGT levels are associated with several hemodynamic parameters, including clinical blood pressure^22–24^, ambulatory blood pressure^25^, heart rate^26^, left ventricular mass index, and intima-media thickness^27^. However, the relationship between urinary AGT excretion and central and local hemodynamics is not fully understood. Our observations confirm that urinary AGT levels are positively and independently associated with aortic blood pressures and pulsatile measures of renal blood flow. To the best of our knowledge, this is the first study to demonstrate that elevated urinary AGT excretion (i.e., intrarenal RAS activity) may play a key role in regulating hemodynamic alterations in the aorta and kidneys.

Previous studies have reported that urinary AGT levels correlate positively with urinary albumin excretion, and negatively with estimated GFR^16,19^. Similarly, the present study revealed that urinary AGT levels positively correlated with the log-transformed urinary ACR (*r* = 0.77) and increased in a stepwise fashion with decline of estimated GFR (Table 2). Although circulating AGT produced and secreted by the liver cannot normally be filtrated across the glomerular basement membrane^13^, basic research has indicated that glomerular injury leads to the excretion of liver-derived (i.e., circulating) AGT into urine and an increase in intrarenal angiotensin II generation^28,29^. These results suggest that glomerular injury may be a trigger of intrarenal RAS activation^10^. However, our stratified analysis based on the presence or absence of albuminuria showed that urinary AGT levels showed a positive correlation with central (aortic) and renal hemodynamic parameters even in individuals with normoalbuminuria (Table S1). Therefore, these findings indicate that elevated urinary AGT may serve as a predicting factor to the alterations in central (aortic) and renal hemodynamics in individuals without glomerular damage.

Urinary L-FABP levels strongly correlated with urinary AGT levels among various parameters (Fig. 2A; Table 4). In addition, the mean urinary L-FABP values increased in the group with lower GFR and higher urinary AGT levels (Fig. 3B). L-FABP is expressed in renal proximal tubular cells and excreted in the urine in response to tubulointerstitial damage^30^. Also, a previous study showed that urinary L-FABP levels strongly correlated with peritubular capillary blood flow directly assessed in living-related kidney transplantations using intravital video analysis^31^. Therefore, our findings suggest that augmented intrarenal RAS activity contributes to the pathogenesis of tubular abnormalities and deteriorates the intrarenal microcirculation. In this context, the results of our study are consistent with basic evidence demonstrating that the expression of renal human L-FABP and urinary human L-FABP levels are increased upon intrarenal RAS activation in human L-FABP chromosomal transgenic mice^32–34^.

It was previously reported that subclinically elevated circulating NT-proBNP levels were associated with an increased risk of developing cardiovascular disease^35^ and CKD^36^ in the general population. Thus, circulating NT-proBNP has been recognized as a representative biomarker of CRS in clinical practice^2,3^. The present study found that increased urinary AGT levels were independent determinants of plasma NT-proBNP levels (Table 3). Moreover, increased plasma NT-proBNP levels associated with reduced renal function were only observed in the group with higher urinary AGT levels (Fig. 3A). A previous study also demonstrated that time course changes in urinary AGT levels on admission and at discharge and readmission were significantly associated with changes in circulating NT-proBNP levels^37^. Therefore, these findings imply that urinary AGT excretion may serve as a useful biomarker for monitoring CRS^2^.

Among five known types of CRS, secondary CRS (type 5) reflects a systemic process that causes simultaneous cardiac and renal damage/dysfunction^3,4^. Several systemic disorders—such as diabetes, amyloidosis, vasculitis, sepsis, and central hemodynamic alterations—have been postulated as potential causes of secondary CRS^3,4,9^. In this study, we found that urinary AGT excretion was a common determinant of central (aortic) and renal hemodynamic alterations. In addition, the results of a previous study showed that increased urinary AGT levels were associated with the incidence of cardiovascular and renal complications in patients with type 2 diabetes and albuminuria^38^. Taken together, these findings suggest that increased intrarenal RAS activity may contribute to systemic conditions leading to secondary CRS.

The present study had several limitations; first, this was a cross-sectional investigation with a relatively small sample size. Thus, we could not address the temporal or causal relationships between urinary AGT levels and central (aortic) and renal hemodynamic parameters. In addition, this study is limited to investigating the association between urinary AGT levels and several CRS surrogate markers. Therefore, the long-term prognostic value of urinary AGT in relation to cardiovascular events and its association with changes in estimated GFR slope remains obscure. Future prospective studies are needed to confirm whether increased urinary AGT excretion can lead to changes in central (aortic) and renal hemodynamics. Additionally, these studies should aim to establish the significant relationship between urinary AGT and not only surrogate markers but also clinically relevant outcomes, such as cardiovascular events and changes in estimated GFR slope. Second, we determined the CKD status via a single assessment of the estimated GFR (< 60 mL/min/1.73 m^2^) and/or urinary ACR (≥ 30 mg/g); however, the diagnosis of CKD usually requires these conditions to have persisted for at least 3 months. Moreover, the etiology of CKD (e.g., glomerulonephritis, diabetic nephropathy, and nephrosclerosis) could not be surveyed in the present study. Third, the association between urinary and plasma AGT concentrations was not investigated in the participants of this study. Previous studies have shown that the level of AGT in renal tissue is significantly lower compared to plasma^13^. This finding suggests that there may be a discrepancy between urinary and plasma AGT concentrations, particularly in individuals without glomerular damage (i.e., individuals without AGT leakage from the blood). Further analyses using a dataset that includes both plasma and urinary AGT concentrations are necessary. Finally, the hemodynamics of the aorta and kidney was not measured simultaneously; still, they were measured in the resting state on the same day in the morning. Thus, the results of this study should be interpreted with caution and confirmed by other studies. Despite these limitations, this study had several strengths; first, it included a comprehensive dataset of biochemical and hemodynamic measurements that may have affected the relationship between urinary AGT levels and central (aortic) and renal hemodynamic parameters. Second, the analysis included patients both with and without CKD, allowing for the comparison of physiological and pathophysiological differences in urinary AGT within a single study.

In conclusion, this study demonstrates that urinary AGT levels are independently associated with several hemodynamic parameters in the aorta and kidneys. Furthermore, higher urinary AGT levels were found to be associated with increased plasma NT-proBNP and urinary L-FABP levels. Collectively, these findings indicate that urinary AGT excretion is a shared determinant of central (aortic) and renal hemodynamics and may lead to a better understanding of the potential role of intrarenal RAS activity in the development of CRS.

## Methods

### Participants

The data in the present study were collected during a voluntary, community-based physical examination conducted in 2018–2019 at the University of Tsukuba. All participants were recruited through local newspaper advertisements, study flyers, homepages, and the Department of Nephrology at the University of Tsukuba Hospital. Two hundred eighty-two participants (66% female) were included in the analysis after excluding participants with young age (< 45 years), missing hemodynamic data, or who did not comply with the study protocol. This study was approved by the Institutional Review Board of the University of Tsukuba and performed in accordance with the guidelines of the Declaration of Helsinki. Written informed consent was obtained from all the participants for study participation.

### Biochemical measurements

Each participant abstained from large meals and vigorous exercise a day before the measurements. Antecubital venous blood samples were collected in the morning following overnight fasting to measure the concentrations of HDL and LDL cholesterols, triglyceride, glucose, hemoglobin A1c, NT-proBNP, creatinine, and cystatin C. These parameters were measured at Tsukuba i-Laboratory LLP using certified methods. The serum FGF23 concentrations were measured using a commercial enzyme-linked immunosorbent assay kit (Kainos Laboratories Inc., Tokyo, Japan). The estimated GFR was calculated using Japanese estimated GFR equations based on standardized serum creatinine or cystatin C levels^39,40^; the average estimated GFR_cr_ and GFR_cys_ values were subsequently used for the analysis.

Urinary concentrations of albumin, β2-microglobulin, L-FABP, AGT, and creatinine were measured using spot urine samples collected in the morning after blood sampling. Among these, the urinary concentrations of albumin and β2-microglobulin were measured using the immunonephelometry and latex agglutination immunoassay methods, respectively. Following the manufacturer’s instructions, urinary L-FABP concentrations were measured using a highly sensitive sandwich enzyme-linked immunosorbent assay (High Sensitivity Human L-FABP ELISA Kit; CMIC Holdings Co., Ltd., Tokyo, Japan)^41^, while urinary AGT concentrations were determined using a human total AGT assay kit (Immuno-Biological Laboratories, Gunma, Japan)^21^. All urinary measurements were reported as ratios relative to urinary creatinine concentrations.

### Hemodynamic measurements

On the same day as the biochemical assessments, several hemodynamic parameters were measured in an environmentally controlled laboratory after adequate rest. Brachial blood pressure and heart rate were assessed using a semiautomated vascular testing device with an electrocardiogram and oscillometric cuffs (form PWV/ABI: Model BP203RPEII; Colin Medical Technology, Aichi, Japan), and applanation tonometry (TU-100; Colin Medical Technology, Aichi, Japan) was used to record the beat-to-beat carotid pressure waveform, which was converted to an aortic pressure waveform using a validated general transfer function (SphygmoCor version 8.0; AtCor Medical, Sydney, Australia) as described in a previous study^42^. Subsequently, the aortic pressure waveform was calibrated using the mean arterial pressure and brachial DBP to determine the aortic systolic and pulse pressures. The aortic incident pressure wave height and augmented pressure adjusted for heart rate of 75 bpm were determined as previously described^43^, and the carotid-femoral (i.e., aortic) PWV—the current gold standard measure of aortic stiffness—was measured using a standard procedure^44^.

The blood flow velocity profiles in the intrarenal segmental arteries of each kidney were recorded using duplex ultrasonography with a 3.5-MHz convex array probe (Noblus C25; Hitachi Aloka Medical Ltd., Tokyo, Japan). Peak systolic and end-diastolic flow velocities were measured with the focal zone set to the depth of the target artery, and the probe insonation angle to the target artery set to < 60°. The renal PI and RI were calculated from these values, as previously described^5^.

### Statistical analysis

Data were expressed as median with interquartile range for continuous data, or frequencies and percentages for categorical data. Group differences according to estimated GFR levels were compared using Kruskal–Wallis nonparametric tests for continuous variables, and the Chi-squared test for categorical variables. For subsequent statistical analyses, variables with skewed distributions were log-transformed to reduce heteroscedasticity and standardized to a normal distribution. Pearson product–moment correlations and stepwise linear regression analyses were used to determine the association of urinary AGT levels with central (aortic) and renal hemodynamic parameters, with adjustment for potential covariates. In the stepwise models, central (aortic) and renal hemodynamic parameters were entered as the dependent variables, while age, sex, BMI, estimated GFR, and medication use—such as renin–angiotensin system inhibitors, calcium channel blockers, diuretics, β-blockers, lipid-lowering medications, and glucose-lowering medications—were entered as the independent variables. Moreover, two-way analysis of covariance was performed to assess the adjusted effects of combined exposure to urinary AGT (categorized according to the median value) and GFR stages on plasma NT-proBNP and urinary L-FABP levels. In the case of a significant F-test for the group effect, the Bonferroni method was corrected for post hoc pairwise comparisons. All statistical analyses were performed using SPSS Statistics software (version 28.0; IBM Inc., New York, USA), and a *P* value < 0.05 was considered statistically significant.

## Supplementary Information

Supplementary Information.

## Data Availability

The datasets generated and/or analyzed during the current study are not publicly available owing to ethical and legal constraints; however, anonymized data are available from the corresponding author upon reasonable request.
