# Supplementary material for: Elevated urinary angiotensinogen excretion links central and renal hemodynamic alterations
Source: Sci Rep. 2023 Jul 17;13:11518. doi: 10.1038/s41598-023-38507-w (PMC10352254; doi:10.1038/s41598-023-38507-w)
Supplement: Supplementary file 1 — Supplementary Information. [file 41598_2023_38507_MOESM1_ESM.docx]

**Elevated urinary angiotensinogen excretion** **links** **central and renal hemodynamic alterations**

Keisei Kosaki^1^, Jiyeon Park^1^, Masahiro Matsui^1^, Takeshi Sugaya^3^, Makoto Kuro-o^4^, Chie Saito^5^, Kunihiro Yamagata^5,6^, Seiji Maeda^1,7^

^1^Institute of Health and Sport Sciences, University of Tsukuba, Ibaraki, Japan

^2^Japan Society for the Promotion of Science, Tokyo, Japan

^3^Division of Nephrology and Hypertension, Department of Internal Medicine, St. Marianna University School of Medicine, Kanagawa, Japan

^4^Division of Anti-aging Medicine, Center for Molecular Medicine, Jichi Medical University, Tochigi, Japan

^5^Department of Nephrology, Institute of Medicine, University of Tsukuba, Ibaraki, Japan

^6^R&D Center for Smart Wellness City Policies, University of Tsukuba, Tokyo, Japan

^7^Faculty of Sport Sciences, Waseda University, Saitama, Japan

**Corresponding author:** Keisei Kosaki, Ph.D.

Institute of Health and Sport Sciences, University of Tsukuba

1-1-1 Tennodai, Tsukuba, Ibaraki, 305-8574 Japan

Tel: +81-29-853-2683; Fax: +81-29-853-2986

E-mail: [kosaki.keisei.gm@u.tsukuba.ac.jp](mailto:kosaki.keisei.gm@u.tsukuba.ac.jp)

**Table S1.** Simple correlation matrix for urinary angiotensinogen and β2-microglobulin in individuals with and without albuminuria

| Variables | Urinary angiotensinogen^*^ | | | | |  | Urinary β2-microglobulin^*^ | | | | |
| --- | --- | --- | --- | --- | --- | --- | --- | --- | --- | --- | --- |
|  | Normoalbuminuria  (n = 198) | |  | Albuminuria  (n = 84) | |  | Normoalbuminuria  (n = 198) | |  | Albuminuria  (n = 84) | |
|  | *r* | *P-value* |  | *r* | *P-value* |  | *r* | *P-value* |  | *r* | *P-value* |
| Aortic systolic blood pressure^*^ | **0.289** | **< 0.001** |  | 0.081 | 0.466 |  | 0.104 | 0.145 |  | 0.083 | 0.452 |
| Aortic diastolic blood pressure | **0.181** | **0.011** |  | 0.066 | 0.552 |  | 0.027 | 0.706 |  | -0.036 | 0.745 |
| Aortic pulse pressure^*^ | **0.254** | **< 0.001** |  | 0.037 | 0.741 |  | **0.145** | **0.042** |  | 0.170 | 0.123 |
| Aortic augmented pressure | **0.284** | **< 0.001** |  | 0.084 | 0.450 |  | **0.211** | **0.003** |  | 0.089 | 0.419 |
| Carotid-femoral pulse wave velocity^*^ | **0.181** | **0.011** |  | 0.081 | 0.463 |  | -0.013 | 0.859 |  | 0.091 | 0.410 |
| Plasma NT-proBNP^*^ | **0.167** | **0.019** |  | **0.253** | **0.020** |  | **0.183** | **0.010** |  | **0.401** | **< 0.001** |
| Urinary L-FABP^*^ | **0.182** | **0.010** |  | **0.583** | **< 0.001** |  | **0.254** | **< 0.001** |  | **0.596** | **< 0.001** |
| Urinary ACR^*^ | **0.439** | **< 0.001** |  | **0.713** | **< 0.001** |  | **0.336** | **< 0.001** |  | **0.341** | **0.002** |
| Renal resistive index^*^ | **0.248** | **< 0.001** |  | 0.181 | 0.100 |  | 0.072 | 0.315 |  | 0.260 | 0.017 |
| Renal pulsatility index^*^ | **0.250** | **< 0.001** |  | 0.179 | 0.103 |  | 0.071 | 0.322 |  | 0.268 | 0.014 |
| Serum FGF23^*^ | 0.037 | 0.601 |  | **0.297** | **0.006** |  | -0.102 | 0.151 |  | 0.200 | 0.069 |

^*^Log-transformed. *r* indicates Pearson's product-moment correlation coefficients. Bold represents statistical significance. The presence of albuminuria was defined as urinary ACR ≥ 30 mg/g. *NT-proBNP* N-terminal pro-B-type natriuretic peptide, *L-FABP* liver-type fatty acid-binding protein, *ACR* albumin creatinine ratio, *FGF23* fibroblast growth factor 23.

**Table S2.** Simple correlation matrix for urinary angiotensinogen and β2-microglobulin in individuals with higher and lower plasma NT-proBNP levels

| Variables | Urinary angiotensinogen^*^ | | | | |  | Urinary β2-microglobulin^*^ | | | | |
| --- | --- | --- | --- | --- | --- | --- | --- | --- | --- | --- | --- |
|  | Lower NT-proBNP  (n = 141) | |  | Higher NT-proBNP  (n = 141) | |  | Lower NT-proBNP  (n = 141) | |  | Higher NT-proBNP  (n = 141) | |
|  | *r* | *P-value* |  | *r* | *P-value* |  | *r* | *P-value* |  | *r* | *P-value* |
| Aortic systolic blood pressure^*^ | **0.297** | **< 0.001** |  | **0.357** | **< 0.001** |  | **0.186** | **0.027** |  | **0.172** | **0.042** |
| Aortic diastolic blood pressure | **0.304** | **< 0.001** |  | **0.248** | **0.003** |  | **0.173** | **0.040** |  | 0.061 | 0.473 |
| Aortic pulse pressure^*^ | 0.124 | 0.144 |  | **0.306** | **< 0.001** |  | 0.107 | 0.207 |  | **0.216** | **0.010** |
| Aortic augmented pressure | 0.131 | 0.122 |  | **0.265** | **0.001** |  | 0.094 | 0.269 |  | **0.165** | **0.050** |
| Carotid-femoral pulse wave velocity^*^ | **0.210** | **0.012** |  | **0.331** | **< 0.001** |  | 0.056 | 0.511 |  | **0.181** | **0.032** |
| Plasma NT-proBNP^*^ | 0.011 | 0.898 |  | **0.377** | **< 0.001** |  | 0.091 | 0.284 |  | **0.397** | **< 0.001** |
| Urinary L-FABP^*^ | **0.478** | **< 0.001** |  | **0.509** | **< 0.001** |  | **0.439** | **< 0.001** |  | **0.559** | **< 0.001** |
| Urinary ACR^*^ | **0.739** | **< 0.001** |  | **0.776** | **< 0.001** |  | **0.424** | **< 0.001** |  | **0.539** | **< 0.001** |
| Renal resistive index^*^ | **0.218** | **0.009** |  | **0.414** | **< 0.001** |  | 0.115 | 0.175 |  | **0.313** | **< 0.001** |
| Renal pulsatility index^*^ | **0.219** | **0.009** |  | **0.413** | **< 0.001** |  | 0.118 | 0.165 |  | **0.320** | **< 0.001** |
| Serum FGF23^*^ | **0.233** | **0.005** |  | **0.340** | **< 0.001** |  | 0.014 | 0.873 |  | **0.256** | **0.002** |

^*^Log-transformed. *r* indicates Pearson's product-moment correlation coefficients. Bold represents statistical significance. The groups of higher and lower plasma NT-proBNP levels were classified based on their median values (53 pg/mL). *NT-proBNP* N-terminal pro-B-type natriuretic peptide, *L-FABP* liver-type fatty acid-binding protein, *ACR* albumin creatinine ratio, *FGF23* fibroblast growth factor 23.
